# Topology Optimization of Periodic Structures Subject to Self-Weight Loading Using a Heuristic Method

**DOI:** 10.3390/ma17225652

**Published:** 2024-11-19

**Authors:** Katarzyna Tajs-Zielińska

**Affiliations:** Faculty of Mechanical Engineering, Cracow University of Technology, 31-155 Cracow, Poland; katarzyna.tajs-zielinska@pk.edu.pl

**Keywords:** optimal design, topology optimization, continuum structures, periodic structures, multi-component structures, design-dependent loads, compliance minimization, Cellular Automata, heuristic methods, local update rule, numerical optimization methods, engineering applications

## Abstract

This paper deals with the actual and challenging process of the optimal design of topologies of periodic structures taking into account the design-dependent loads. The topology formulation used in this paper minimizes the compliance value of the structure and is subject to a total volume constraint while maintaining a periodic pattern and self-weight load. This combination represents a promising and original contribution to the field of ongoing research, although it is not yet widely recognized. This paper aims to fill this gap by presenting the first results of numerical optimization tests. The redistribution of material within a design domain is governed by the rules of Cellular Automata, a locally oriented optimization tool that can be applied to all types of structural optimization, including topology optimization. The technique has been demonstrated by numerical tests on two- and three-dimensional examples. The calculations were performed for different types of periodic schemes. The optimized structures did not show the checkerboard effect or the presence of residual gray elements in the final topologies. The strategy used in this paper ensures connectivity between periodic subdomains without imposing additional conditions on the algorithm.

## 1. Introduction

The demand for sustainable, durable, and eco-friendly engineering structures is a fundamental requirement for modern design processes. In order to achieve this goal, efficient design tools must be implemented, and innovative computational methods must be applied at the conceptual stage of the project. One of the most powerful tools in modern design, including mechanical engineering and civil engineering applications, is topology optimization. The idea of topology optimization is to find the optimum distribution of material within a defined area, according to the criteria defined. The new layout of the material should minimize a defined cost function and satisfy previously imposed constraints. The most common formulation of topology optimization is to minimize the compliance value of the structure subject to a total volume of the exploited material. Meanwhile, less common but still important issues, like manufacturing constraints and/or symmetry and pattern repetition, can significantly influence useful properties of the final design. Following this trend, this paper investigates a simple method for topology optimization of structures under periodicity constraints with particular emphasis on the structures under design-dependent loads. The optimization technique chosen is a heuristic method called Cellular Automata (CA) because of its simplicity and versatility. Cellular Automata are mathematical models consisting of a lattice of cells, the states of which evolved from local rules that mimic the global behavior of the system. The idea was introduced by von Neumann and Ulam in the 1940s. In the mid-1990s, Inou et al. [1] proposed an application of the concept as an optimization tool. The method has been found attractive for size optimization [2,3] and topology optimization, e.g., [4,5,6]. The standard compliance minimization problems (e.g., [7]), problems with stress and displacement constraints (e.g., [8]), reliability-based topology optimization (e.g., [9]), optimization of multi-material structures (e.g., [10,11]), and topology optimization of energy absorbers [12] can be found in the literature. The above list can be extended by applying the method to uncommon examples, such as periodic structures, under design-dependent loads. In particular, the additional challenge for the algorithm is the constraint on the repetitiveness of the resulting material layout. This repetitiveness is defined by the periodic constraints executed by averaging the compliance within the basic macrocells. Furthermore, the rarely discussed and challenging application of self-weight loading makes the solutions obtained more practical and realistic. The idea presented in this paper is unique in the literature as far as self-weight loading is concerned.

Topology optimization (TO) is a powerful tool for the optimization of structural layout that aims to find the best material distribution within a design domain while satisfying mechanical, geometrical, and manufacturing specifications. Since TO was developed, it has been utilized in almost all fields of engineering and science. The basis of TO, methods, and techniques can be found in numerous papers, such as [13,14].

One of the challenges in applying topology optimization in industrial settings is the tendency to design complex layouts of structures, which can significantly increase construction costs, sometimes overtaking cost savings gained using reduced materials. The solution to that could be creating periodic structures that would allow individual elements of a structure to be manufactured in a recurring and, therefore, cost-effective way. By designing pre-manufactured modular sections, topology optimization can help reduce the environmental impact of industry and construction by enabling the creation of lightweight and robust structures in a manufacturing and assembly-friendly way. The application of periodic structures, in certain cases, can be encouraging because of their unique advantages, illustrated by [15,16,17,18]. Furthermore, the complexity of the structures can be reduced by the introduction of repetitive patterns. The application of periodic systems enables the optimization of larger-scale problems that would otherwise be intractable with low computational costs. The use of periodic and repeatable structures is consistent with the principles of architectural design, which is a significant advantage in this field, particularly given the increasing use of topological optimization as a design tool in both architecture and civil engineering. Periodic topology optimization was developed in parallel with TO, primarily to deal with the optimal design of effective material properties, i.e., at the microstructure level [19,20,21,22]. This approach is examined in a wide range, as the designing of cellular materials and an extended overview of this topic can be found in [23,24]. The early works on the designing of the periodic structures at the macro level are referenced in [25,26,27]. The relations between the optimal design of material microstructures and periodic macrostructures are described in [28]. Among many inspiring papers, one can find those addressing various types of topology optimization problems like multi-objective periodic topology optimization [29], robust topology optimization for periodic structures [30], dynamic problems, like topology optimization of periodic composites with dissipative materials [31], or the topology optimization of periodic structures for the crash and static load cases [32]. In [18], the authors present the periodic topology optimization for structures with orthotropic materials, showing that these kinds of materials resist deformation and reduce von Mises stress in periodic structures more efficiently than isotropic materials. Topology optimization for periodic multi-component structures with stiffness and frequency criteria with the inclusion of investigation interfacing connections between periodic components is discussed in [33]. The periodicity constraints and the configuration of periodic subdomains have a significant influence on the value of the final results. To minimize that negative effect, a multi-pattern control was introduced in [34]. A very interesting aspect of pattern repetition in TO is discussed in [35,36,37], where the pattern gradiation is considered. In [36], the pattern can be mapped on regular or irregular design domains. Periodic topology optimization is traditionally realized by imposing periodic constraints on the global structure. The idea of [38] combines a topology optimization algorithm with biologically inspired pattern formation, offering an alternative to the most common approach.

Periodic topology optimization is still in the developmental phase, and there is still room for further research to complement the papers published thus far. This paper tries to fill the gap in the mentioned research area by utilizing a heuristic algorithm as a generator of optimal topologies of periodic structures considering self-weight loading. The combination under consideration appears to be a viable and novel contribution to the ongoing investigation, although it is not yet widely recognized. This paper contributes to the development of the research by presenting the initial results of numerical optimization tests of the formulated, original problem of periodic topology optimization of structures under a self-weight loading.

This paper is organized as follows. Section 2 introduces the concept of Cellular Automata with flexible rules. Section 3 presents an approach to topology optimization that combines a Cellular Automaton model with the ANSYS software suite to provide an efficient analysis tool. This section also demonstrates the applications of this methodology through the analysis of selected numerical optimization examples of periodic structures under self-weight loading. Section 4 discusses the numerical results, and Section 5 concludes this paper with a summary of the test results.

## 2. Straightforward Method of Cellular Automata Addressed to the Topology Optimization of Periodic Structures

### 2.1. Cellular Automata Basis

Heuristic methods have been developed to solve optimization problems quickly, effectively, and efficiently. Heuristics such as the Genetic Algorithm, Simulated Annealing, Ant Colony Methodology, Particle Swarm Optimization, Probabilistic Learning, Big Bang–Big Crunch Algorithm, Hunter–Prey Optimization Algorithm, and others [39,40,41] are proving attractive for solving optimization problems, including topology optimization. Since the first application of Cellular Automata (CA) to topology optimization [1], this method has been added to the list of effective tools mentioned above. The application of the Cellular Automata method to topology optimization requires, similar to the Finite Element Method, results in the discretization of the design domain under consideration into a grid of cells (elements). The exchange of information between neighboring cells is the principle of the Cellular Automaton. Each cell is represented by a certain number of states. These states are updated from iteration to iteration by applying a local update rule that uses information from both the given cell and its neighbors. A characteristic feature of Cellular Automata is the local nature of the information exchange, which is realized simultaneously for all cells. Individual algorithms usually differ in their local update rules. More information on the main concept and theoretical aspects of CA research can be found in [42]. This paper discusses the use of CA as an optimization tool in engineering. In engineering optimization applications, the classical Cellular Automaton consists of five basic features: discrete one-, two-, or three-dimensional lattice of cells, assumption of homogeneity, i.e., all cells of the grid are equivalent (discussion of this assumption can be found in [43]), discrete states (each cell can be in one of a finite number of possible discrete states related to design variables), local update rule applied simultaneously for all cells and contained only with the local environment of the cell, and discrete dynamics (in each discrete unit of time, i.e., iteration, the state of each cell is updated according to a local rule using information from the immediate neighborhood of the cell).

The most commonly implemented neighborhood types are the von Neumann, Moore, and Radial types described in detail along with other possibilities in [44]. In the current study, the Moore type was adopted (neighboring cells share the vertices with the central cell). The boundary conditions must be defined according to the type of neighborhood.

When forming a neighbor for cells located at the boundary of the design domain, cells outside the design domain must be considered. The most common approach is to assume that a specific value of the quantity characterizing states of boundary cells is zero (specified boundary conditions). However, it is also possible to define reflecting, adiabatic, or periodic boundary conditions. In the case of periodic boundary conditions, the neighbors of the boundary cells are those cells that are adjacent to the opposite edge of the mesh. This type of boundary condition is reasonable for periodic structures.

### 2.2. Periodic Topology Optimization Using Local Rules of Cellular Automata

The periodicity of the structure is achieved by partitioning the design domain into subdomains in both the horizontal and vertical directions, with periodic constraints being imposed (i.e., periodic topology configuration, as shown in Equation (3)). The periodic topology optimization problem is formulated in this paper as a minimization of structure compliance (Equation (1)) subject to a total volume constraint (Equation (4)). The minimization of the objective function (i.e., the structure compliance) results in the minimization of the strain energy stored in the structure to carry out the applied loads.

(1) $$minimize\;\;\;\;\;\;U(d_{ij})=\sum_{i=1}^{N}\sum_{j=1}^{M}{{(d}_{ij})}^{p}u_{ij}^{T}k_{i}u_{ij}$$

(2) $$subject\;to\;\;\;\;\;\;0<d_{min}\leq d_{i,j}\leq 1,$$

(3) $$d_{i1}=\ldots =d_{ij}=\ldots =d_{iM},$$

(4) $$V\left(d\right)=\kappa V_{0}=\sum_{i=1}^{N}\sum_{j=1}^{M}d_{ij}v_{ij},$$

where **u***_ij_* represents the displacement vector of the *i*-th element in a *j*-th subdomain, **k***_i_* stands for the *i*-th element stiffness matrix, and *d_ij_* is material relative density (design variables indicating the presence of material within individual elements). To represent voids, the design variable is set to 0 (or a very low value of *d_ij_* = 0.001). Consequently, *d_ij_* = 1 is used for elements with full material. The relative density is a continuous variable and varies continuously between *d_min_* and 1. Intermediate values, also known as the ‘grey elements’ that appear during the optimization process, are consequently removed from the solution. The minimal value *d_min_* is imposed on the design variables (Equation (2)) to avoid singularity during the finite element analysis. All quantities are defined for *N* elements and *M* periodic subdomains. The total volume constraint is defined by Equation (4), where *V*_0_ denotes the volume of the material for the whole design domain, *κ* is a volume fraction and defines the amount of the material in the optimized structure, and *v_ij_* is the volume of a unit cell/element. Equation (4) represents the equality constraint, i.e., the volume of the utilized material remains constant throughout the optimization process. The periodicity constraint imposed on the design variables by Equation (3) ensures the desired repetitiveness in the structure. The discretization of each subdomain is achieved using a regular, identical mesh, with the result that the corresponding elements are positioned identically in each subdomain. The design variables described by the corresponding elements in the *M* subdomains are equal, making it necessary to evaluate them simultaneously for each subdomain. It should be noted that the constraint expressed by Equation (3) (i.e., periodicity and the subdomain configuration) affects the final compliance value, as discussed in Section 3 of this paper or in [32,45].

The assumed formulation requires a definition of the material representation. In the case of design-dependent loads (such as the structural self-weight), this can be performed by the modification of the SIMP approach [46]. The Young modulus *E_ij_* and material density *ρ_ij_* of elements must be modeled as a function of relative density *d_ij_*, as shown in Equations (5) and (6) as follows:

(5) $$E_{i,}=d_{ij}^{p}E_{0},$$

(6) $$\rho_{ij}=d_{ij}^{p}\rho_{0}.$$

The quantities *E*_0_ and *ρ*_0_ stand for the modulus of elasticity and the material density of a solid material, and *p* is a penalization power of intermediate densities; typically, *p* = 3. To prevent a parasitic effect [47], the extension of the standard SIMP method is essential; therefore, the updated model of the material density *ρ_ij_* of each element is defined according to Equation (6). As discussed in Section 4, consideration of the self-weight loading in the topology optimization process can have a major impact on the final layout of the structure. The aforementioned impact is explored in detail in the early studies on self-weight loading in topology optimization [46]. This challenging and important field of research is reviewed in [47,48,49,50] as an element that brings novelty to the consideration of the optimal design of structures under design-dependent loads.

The formulated problem can be solved by classical methods or the efficient Cellular Automata method. When using CA, the local update rule plays a key role in the optimization process. It defines how information about the values of the state variables, obtained as a result of solving the structure analysis, is transmitted to the cells. For this paper, the rule proposed and examined in [51] was chosen as the update scheme because of its advantages. The main idea of the concept is based on the flexible update process that determines the new value of the cell design variable under consideration based on the compliances of neighboring cells.

Let us define the update rule as performed in Equations (7) and (8). The design variables are updated based on the information *F*(*i*,*j*) gathered from the central cell and the sum of the information *F*(*k*) from the *S* cells forming a neighborhood as follows:

(7) $$d_{ij}^{new}=d_{ij}+{∆d}_{ij},$$

(8) $$∆d_{ij}=\left[F\left(i,j\right)+\sum_{k=1}^{S}F(k,j)\right]\frac{m}{s+1},$$

where *m* = 0.2 is a move limit selected in the numerical tests.

The function *F*(*i*,*j*) is defined for all cells and is related to the sorted element compliance values. If the elements are arranged in ascending order according to their compliance values, it is possible to ascertain the number of elements exhibiting the lowest *N*_1_ and highest *N*_2_ compliance values. If the compliance value of the cell is sufficiently small (or sufficiently large), a constant value of *C_α_*_1_ is assigned (or *C_α_*_2_, respectively), as illustrated in Equation (9).

(9) $$F\left(i,j\right)=\left\{\begin{array}{c} C_{\alpha 1}\;\;if\;\;i<N_{1}\\ f\left(i,j\right)\;\;\;if\;\;N_{1}\leq i\leq N_{2}\\ C_{\alpha 2}\;\;\;if\;\;i>N_{2} \end{array}\right.$$

In the original idea [51], *C_α_*_1_ = −*C_α_* = −1 and *C_α_*_2_ = *C_α_* =1. It is proposed to introduce a monotonically increasing function presented in Equation (10) for the intermediate interval *N*_1_ ≤ *i* ≤ *N*_2_ as follows:

(10) $$f(i,j)=C_{\alpha }\frac{tanh\left[\beta (\frac{i-N_{1}}{N_{2}-N_{1}}-\frac{1}{2})\right]}{tanh\;(\frac{1}{2}\beta )}.$$

The purpose of the brief presentation of the rule posted above is to familiarize the reader with the main concept underlying the methodology. However, a comprehensive understanding of the adopted scheme, including a detailed examination of the adjustments of the parameters of the method and numerical tests, can be obtained from the introductory paper [51].

Let us look at the adapted parameters used to optimize the periodic topology. The benefit of the adaptation was the identification of a constant *β* that was suitable for all examples. The value of *β* was set at 0.01 following a process of numerical analysis. This value allows us to approximate the function *f*(*i*,*j*) to a relatively simple form of linear expression (Equation (11)) as follows:

(11) $$f(i,j)=\frac{2i-N_{2}-N_{1}}{N_{2}-N_{1}}.$$

Furthermore, the flexibility of the method allows us to improve the performance of the algorithm for specific problems. As indicated by the results of the numerical tests, the constant *C_α_* was transformed into two values: *C_α_*_1_ = −1.125 for the *N*_1_ elements with the lowest compliance values and *C_α_*_2_ = −0.125 for the *N*_2_ elements with the highest compliance values. This procedure allowed for a reduction in the number of tests performed for each 2D and 3D structure and assumed one value of the parameters for all examples. Therefore, the final form of the *f*(*i*,*j*) function is (see Equation (12)) as follows:

(12) $$f(i,j)=\frac{i-N_{2}-N_{1}}{N_{2}-N_{1}}$$

The proposed modification is used for *N*_1_ = 0.1 *N* and *N*_2_ = 0.9 *N*, where *N* is the number of cells/elements; reducing the interval [*N*_1_, *N*_2_] leads to an acceleration of the convergence of the algorithm [51].

The constructed design rule is implemented to the numerical algorithm combined with the Ansys package as an effective analysis tool. The pseudo-code of the algorithm is given below (Algorithm 1). Concerning the optimization procedure, the sequential approach has been adapted. This implies that for each iteration, the structural analysis performed for the optimized element is followed by the local updating process.
**Algorithm 1.** CaptionGET input data SELECT *N*_1_ and *N*_2_ values SET initial values of design variables SELECT neighborhood type ASSIGN neighbors to each element SELECT move limit *m* DO UNTIL stopping criteria are met         PERFORM structural analysis         IMPORT data from structural analysis         FOR all elements                   IMPOSE periodicity constraints                   CALCULATE local compliances U(*d_i_*)         END FOR         SORT compliances in ascending order         BUILD C(*i*) function         FOR all elements                   UPDATE design variables *d_i_*         END FOR         IMPOSE volume constraint END DO DISPLAY results

The initial values of the design variables are uniformly distributed throughout the design domain to satisfy the volume constraint. As mentioned above, the Moore type was adopted for two- and three-dimensional examples. The stopping criterion has been defined as the improvement of the objective function value for subsequent iterations below 1‰.

**Figure 1:**
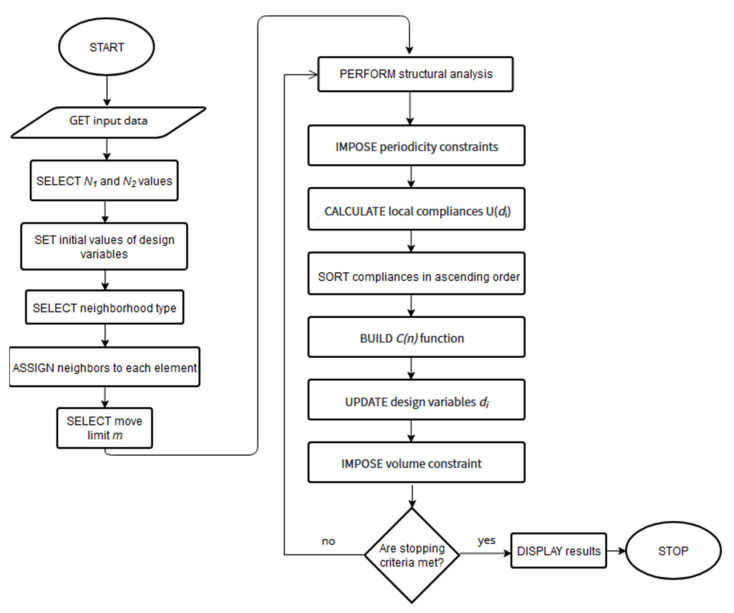
The flowchart of the topology optimization algorithm.

A global volume constraint is implemented in each iteration step following the application of local update rules to all elements. The generated topologies preserve a specific volume fraction of a solid material throughout the whole optimization process by rescaling the values of the design variables. The flowchart of the methodology is presented in Figure 1.

## 3. Numerical Examples of the Topology Optimization of Periodic Structures Considering Self-Weight Loading

The aim of this paper is to present the application of the heuristic method to the design of optimized periodic topologies of structures, with particular attention paid to self-weight loading. In the initial phase of the analysis, the non-periodic structure is subjected to an external concentrated load, and subsequently, the self-weight is included in the investigation. In the second part of the Section, the periodicity is additionally imposed on the final topologies. That combination is (to the best of the author’s knowledge) uncommon in the existing literature on the subject; therefore, some initial findings on the subject are presented. Further research is required to complete and develop the investigation.

Topology generation was performed using the in-house Fortran code, which was executed as a standalone program. An optimization module was combined with a professional system, ANSYS, which is responsible for the structural analyses. The design variables updated by the optimization module serve as the input data for the analysis model built in Ansys Parametric Design Language (APDL).

### 3.1. Topology Optimization of the Two-Dimensional Example Considering Self-Weight Loading

**Figure 2:**
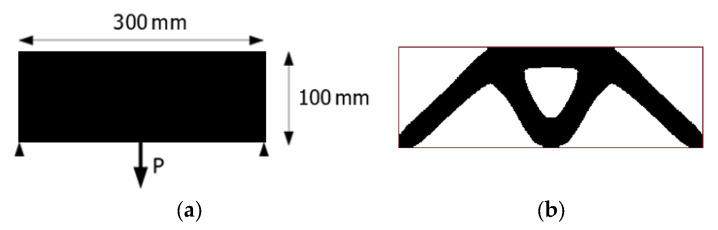
Example 1: (**a**) initial structure, applied load, and supports; (**b**) final topology for applied load and volume fraction 0.4 (no periodicity, no self-weight, P = 100 N, final compliance 17,936 Nmm). The red line shows an initial design space for convenience.

As the first illustration of the proposed idea in this paper, the plain structure presented in Figure 2a with applied load and supports is selected. The structure is divided into *N* = 30,000 (300 × 100) discrete four-node elements arranged in a regular lattice. Each finite element is equivalent to the cell of the Automata. The Young modulus of the solid material equals E_0_ = 10 MPa, whereas the Poisson ratio is ν = 0.35 and material density is ρ_0_ = 1000 kg/m^3^. The assumed volume fraction is *κ* = 0.4, so the values of the initial design variables are set to 0.4 to satisfy the volume constraint. The minimal value of the design variable is set to *d_min_* = 0.001. The penalization power *p* in the SIMP approach is equal to 3, and an allowable maximal move limit *m* is set to 0.2. The parameters *N*_1_ and *N*_2_ responsible for the convergence of the method are equal to *N*_1_ = 0.1 *N* and *N*_2_ = 0.9 *N,* although it is possible to change their values during the iteration process in order to speed it up.

**Figure 3:**
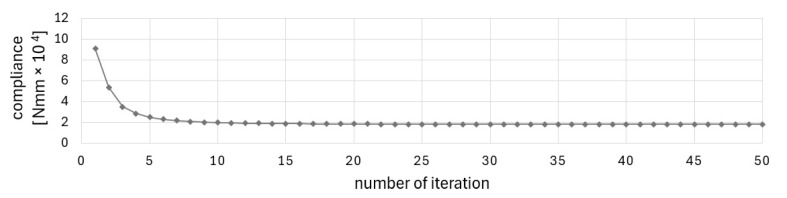
Compliance history for example 1.

**Figure 4:**
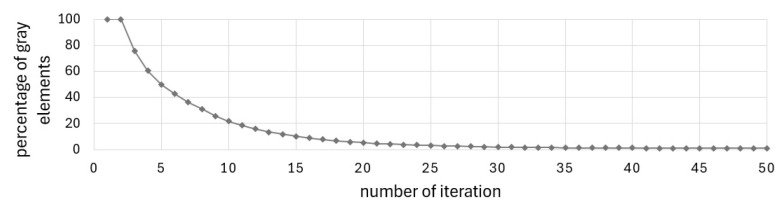
Percentage of gray elements at each iteration step for example 1.

The final topology presented in Figure 2b was achieved at the 27th iteration when the stopping criterion was the improvement of the objective function value below 1‰. Figure 3 and Figure 4 illustrate the iteration process of up to 50 iterations for convincing. Figure 4 shows the change in the percentage of gray elements at each iteration step and the rapid elimination of gray elements.

**Figure 5:**
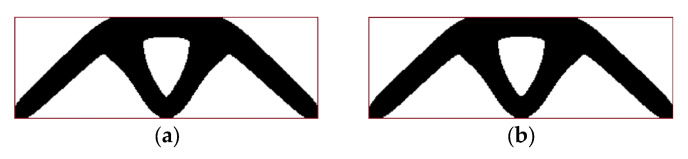

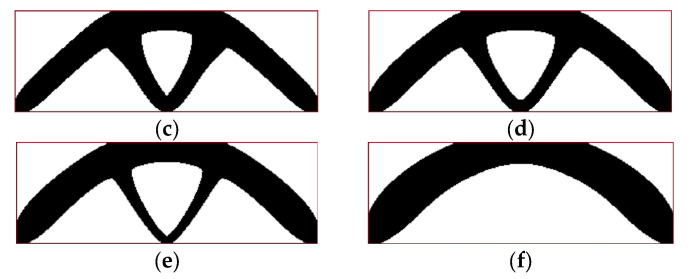
Topologies for example 1 (red line shows an initial design space for convenience): (**a**) applied force equals P = 1000 N and self-weight (final compliance: 2,014,111 Nmm); (**b**) applied force equals P = 500 N and self-weight (final compliance: 571,548 Nmm); (**c**) applied force equals P = 100 N and self-weight (final compliance: 50,045 Nmm); (**d**) applied force equals P = 20 N and self-weight (final compliance: 13,476 Nmm); (**e**) applied force equals P = 10 N and self-weight (final compliance: 10,240 Nmm); (**f**) self-weight only (final compliance: 6758 Nmm).

It is well known that the final topology (Figure 2b) does not depend on the value of the applied load. This rule does not apply in the case of design-dependent loads, such as self-weight. An illustration of this phenomenon can be observed in Figure 5, where, apart from the concentrated force P = 1000 N (Figure 5a), P = 500 N (Figure 5b), P = 100 N (Figure 5c), P = 20 N (Figure 5d), P = 10 N (Figure 5e), and P = 0 N (Figure 5f), the gravitational acceleration 9.81 m/s^2^ is considered.

The optimization results for a combination of design-independent external and self-weight loads depend on the ratio of the load values. When the ratio is sufficiently high (and the external load is of a sufficiently high value), the self-weight has no considerable influence on the final layout of the structure.

### 3.2. Topology Optimization Considering Periodicity Without Self-Weight Load

**Figure 6:**
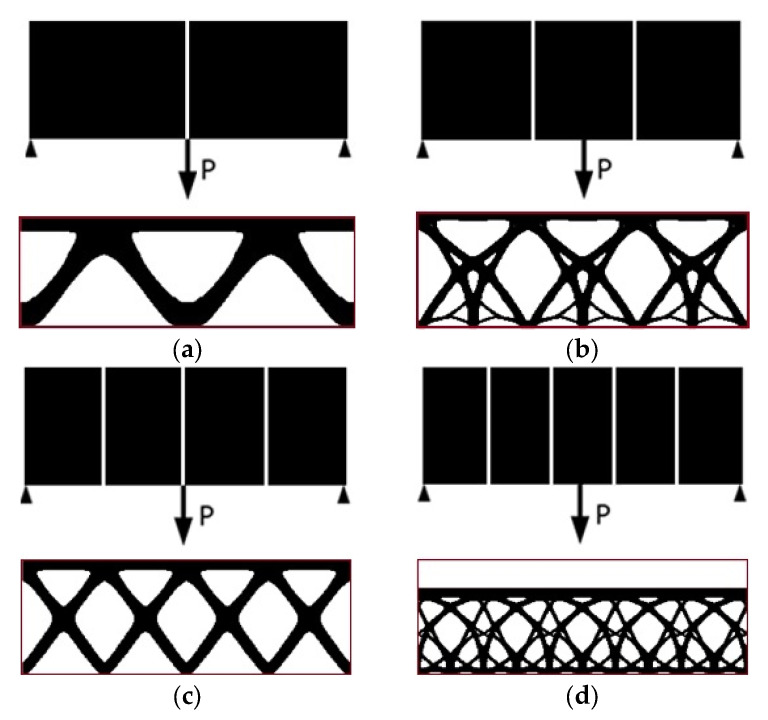
Periodicity schemes and final topologies for example 1 considering concentrated, external load P only (red line shows an initial design space for convenience): (**a**) periodicity scheme I: 2 subdomains (final compliance: 21,814 Nmm); (**b**) periodicity scheme II: 3 subdomains (final compliance: 35,710 Nmm); (**c**) periodicity scheme III: 4 subdomains (final compliance: 30,829 Nmm); (**d**) periodicity scheme IV: 5 subdomains (final compliance: 37,199 Nmm).

The periodicity schemes, including minor differences between them, have a significant effect on both the resulting optimized structure and its final compliance. A comprehensive examination of these facts can be found in [32]. In reaching a preliminary determination, the periodicity scheme for example 1 evolved in the horizontal direction only (the material data and algorithm parameters used in the calculations are the same as in Section 3.1). The periodicity schemes contain two, three, four, or five subdomains. The final topologies for external load P = 100 N are presented below (Figure 6a–d)—primarily without self-weight loading.

**Figure 7:**
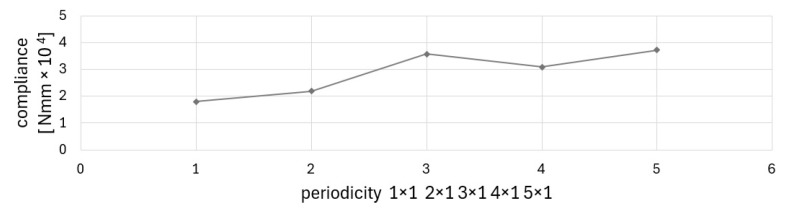
Final values of compliances for example 1 for assumed periodicity schemes.

The impact of the number of subdomains and configuration of periodicity on the final results is illustrated in Figure 7. The resulting topologies are very different for two, three, four, or five subdomains. The value of the final compliance depends on the number of subdomains. The complexity of topologies varies considerably when the force is applied at the center of the subdomain edge (three and five subdomains) in comparison to when the force is applied at the corner of the subdomain (two and four subdomains). This illustrates the significance of the subdomain scheme in terms of its design.

The value of the final compliance is typically higher when there are a greater number of subdomains, which is associated with more stringent restrictions on the distribution of material. It is noteworthy that the value of compliance increases nonmonotically with the number of subdomains (see Figure 7). The same effect is also observed in [32]. This may lead to the conclusion that the optimal balance between periodicity schemes can be achieved to benefit from periodicity and a small value of compliance.

### 3.3. Topology Optimization Considering Periodicity and Self-Weight Loading

The challenge of the optimal design of structures under design-dependent loads (such as the aforementioned structural self-weight, surface pressure, thermo-elastic loads, or centrifugal loads) lies in the variable nature of loading during the optimization process, i.e., location, direction, and magnitude of the loads change with the evolving topology of the structure.

The first step in the investigation of the self-weight loading when considering periodic structures is to check the optimized topologies by considering only design-dependent loads with no external load (P = 0 N). The material data and algorithm parameters used in the calculations are the same as in Section 3.1.

**Figure 8:**
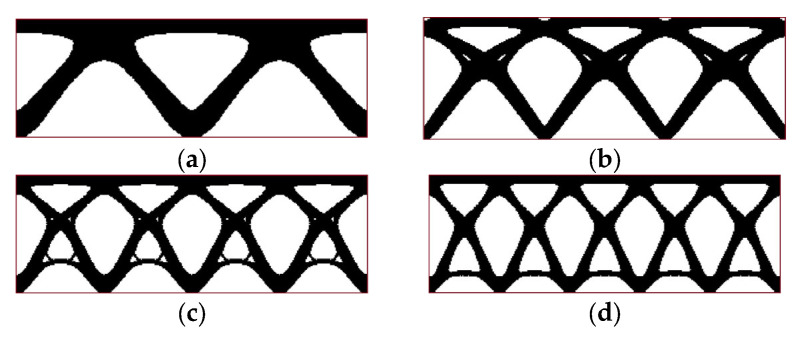
Final topologies for example 1 considering self-weight only (red line shows an initial design space for convenience): (**a**) periodicity scheme I: 2 subdomains (final compliance: 10,968 Nmm); (**b**) periodicity scheme II: 3 subdomains (final compliance: 14,514 Nmm); (**c**) periodicity scheme III: 4 subdomains (final compliance: 17,316 Nmm); (**d**) periodicity scheme IV: 5 subdomains (final compliance: 18,390 Nmm).

Figure 8 presents the final topologies for four periodicity schemes of example 1 proposed in Section 3.2. Self-weight is the only load imposed on the periodic structure.

**Figure 9:**
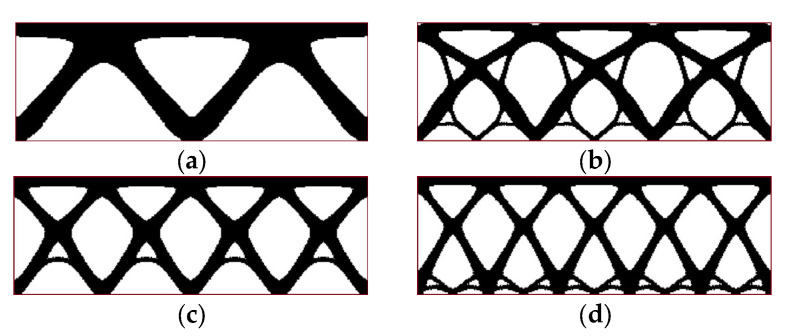
Final topologies for example 1 considering self-weight and external load P = 30 N (red line shows an initial design space for convenience): (**a**) periodicity scheme I: 2 subdomains (final compliance: 20,614 Nmm); (**b**) periodicity scheme II: 3 subdomains (final compliance: 29,733 Nmm); (**c**) periodicity scheme III: 4 subdomains (final compliance: 31,302 Nmm); (**d**) periodicity scheme IV: 5 subdomains (final compliance: 34,704 Nmm).

**Figure 10:**
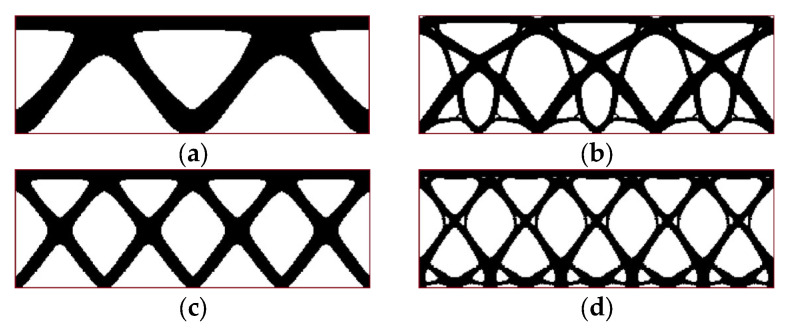
Final topologies for example 1 considering self-weight and external load P = 100 N (red line shows an initial design space for convenience): (**a**) periodicity scheme I: 2 subdomains (final compliance: 58,059 Nmm); (**b**) periodicity scheme II: 3 subdomains (final compliance: 90,058 Nmm); (**c**) periodicity scheme III: 4 subdomains (final compliance: 83,266 Nmm); (**d**) periodicity scheme IV: 5 subdomains (final compliance: 107,902 Nmm).

To complete the investigation, the final topologies obtained for four periodicity schemes for example 1 are presented, but this time, the combination of self-weight and external load is applied. The value of P = 30 N and P = 100 N were selected. Figure 9 presents the final topologies for P = 30 N and Figure 10 for P = 100 N.

**Figure 11:**
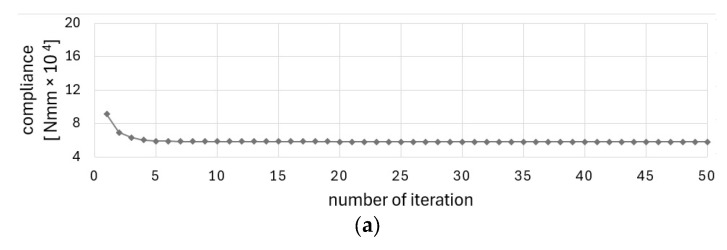

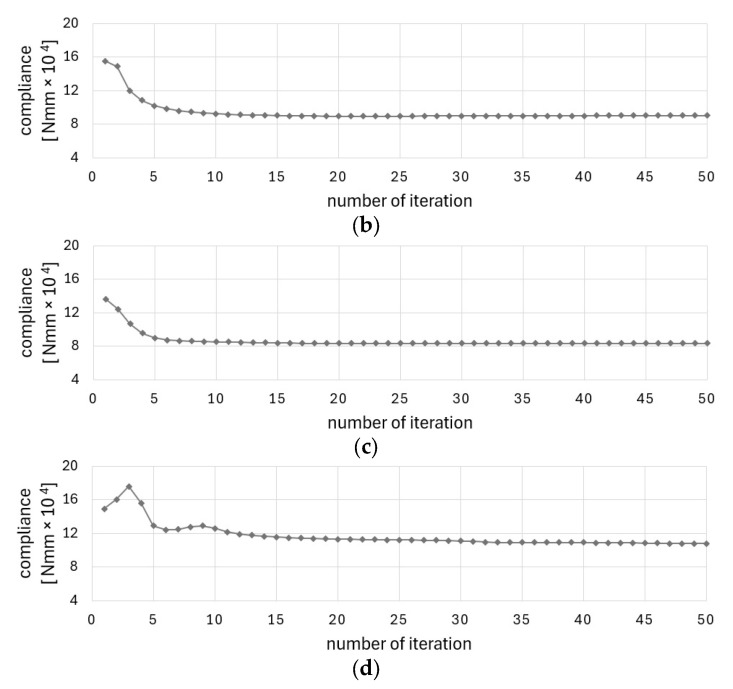
Compliance history for example 1 with five subdomains considering self-weight and external load P = 100 N: (**a**) periodicity scheme I: 2 subdomains; (**b**) periodicity scheme II: 3 subdomains; (**c**) periodicity scheme III: 4 subdomains; (**d**) periodicity scheme IV: 5 subdomains.

The convergence of the solution for the last case (external load P = 100 N and self-weight) is illustrated in Figure 11a–d for all periodicity schemes.

The fundamental criterion for structures formed of components is the interconnection between optimized subdomains. The strategy employed in this paper provides connectivity without the imposition of any additional conditions on the algorithm. The application of periodic boundary conditions at the level of each subdomain can secure the continuity of topology at component boundaries.

### 3.4. Engineering Example of Topology Design Including Self-Weight and Periodicity Scheme

**Figure 12:**
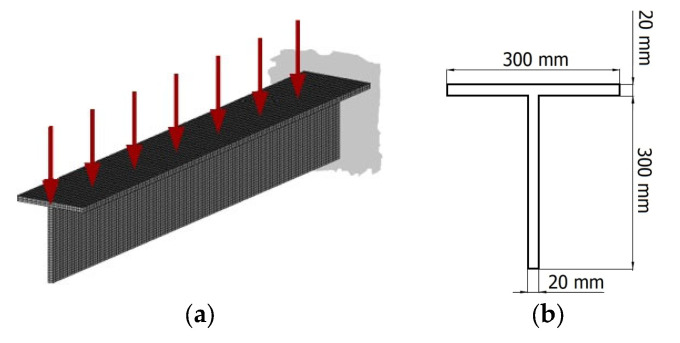
Cantilever T-beam: (**a**) initial structure, applied load, and supports; (**b**) cross-section of the beam.

The engineering example of topology optimization is addressed to a periodic 2 m long cantilever T-beam (see Figure 12). The T-beam is loaded by the distributed force P = 10 MN/m. The Young modulus of the solid material equals E_0_ = 200 MPa, while the Poisson ratio is ν = 0.3 and material density is ρ_0_ = 7500 kg/m^3^. The structure is discretized with *N* = 12,000 eight-node elements in a regular lattice. The assumed volume fraction is κ = 0.4, so the values of initial design variables are set to 0.4 to satisfy the volume constraint. The minimal value of the design variable is set to *d_min_* = 0.001. It is assumed that the flange is a nonoptimized region and only the web is under optimization. Half of the structure is considered according to symmetry. The penalization power *p* in the SIMP approach equals 3, and an admissible maximal move limit *m* is set to 0.2. The parameters *N*_1_ and *N*_2_ responsible for the convergence of the method are equal to *N*_1_ = 0.1 *N* and *N*_2_ = 0.9 *N.*

**Figure 13:**
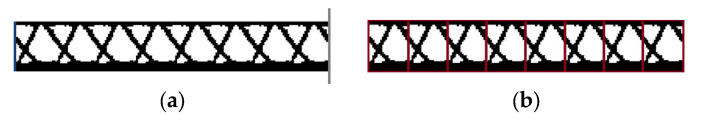
The cantilever T-beam results: (**a**) front view with the closed left end marked in dark blue and the support on the right side marked in gray; (**b**) front view with subdomain boundaries marked in red.

The optimization process started with design variables equal to 0.4 due to the assumed value of the volume fraction. The initial value of the compliance in this case was found to be equal to 9.364 × 10^8^ Nmm. The final compliance reached the 6.575 × 10^8^ Nmm value for the final topology illustrated in Figure 13.

**Figure 14:**
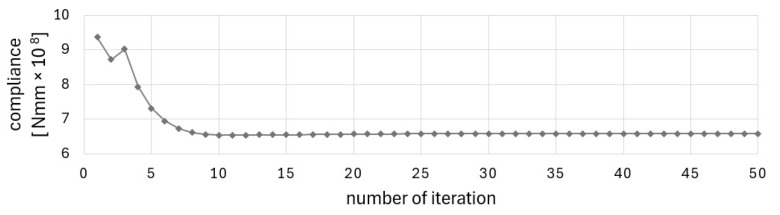
Compliance history for the cantilever T-beam.

**Figure 15:**
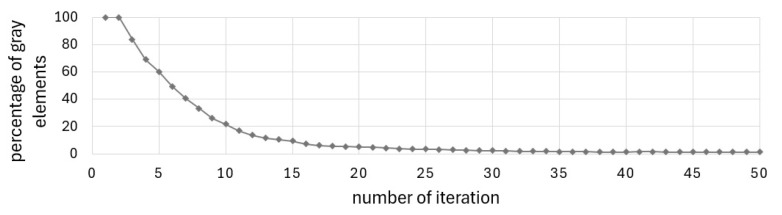
Percentage of gray elements at each iteration step for the cantilever T-beam.

The solution was found in 38 iterations. The stopping criterion was the improvement of the objective function value below 1‰. Figure 14 and Figure 15 illustrate the iteration process of up to 50 iterations for convincing.

The defined complex problem is solved without gray elements in the solution, as illustrated in Figure 15, showing the change in the percentage of gray elements in each iteration step.

**Figure 16:**
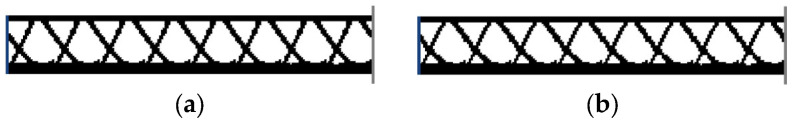
The cantilever T-beam results: (**a**) front view: only self-weight is considered; (**b**) front view: only distributed load P is considered.

The considered example was investigated in the case of self-weight loading without the external distributed load P (Figure 16a) and when the load P was only load acting (Figure 16b).

The resulting topologies are found to be highly similar, which is an unusual outcome. However, it is possible to verify that the resulting topologies are also similar for the structure considered without periodicity.

The edge segments of the T-structure exhibit a shape that is not substantiated from a practical, engineering perspective (unsupported pins—see, e.g., Figure 13a). This is a consequence of the interconnection between optimized subdomains imposed by intermediate positions of repetitive components.

**Figure 17:**
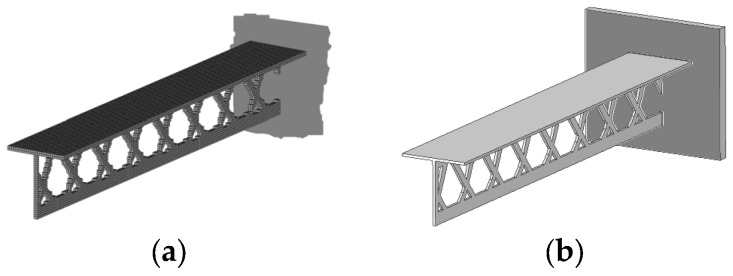
The cantilever T-beam results after redesigning: (**a**) isometric view of the mesh; (**b**) isometric view of the CAD model.

As is the case with the results of topological optimization, an engineering interpretation is required to provide an accurate representation of this artifact in the final design. Figure 17a presents the 3D isometric view of the redesigned topology of the structure considering the rational use of the closed edge segment. Figure 17b presents a CAD model of the considered T-structure.

## 4. Discussion of the Results

The Cellular Automata algorithm, which has been adopted for use in this project, has been found to be an effective tool for structural optimization of periodic structures and the structures under design-dependent loads. The technique studied in [51] was found to be both good converging and versatile. The method was selected for the originally formulated problem due to its simplicity, versatility, and good convergence. The good convergence is understood here as a relatively small number of iterations needed to satisfy the convergence criterion, i.e., the improvement of the objective function value in adjacent iterations is below 1‰. As can be seen in the iteration history diagrams (Figure 3, Figure 11 or Figure 14), the iteration process stabilizes after a few first iterations, and the solution is very close to the optimized results. The algorithm does not require any special filtering strategy to avoid the checkerboard effect and eliminate the gray elements from the final topology. The local update scheme is based on the information gathered within a cell neighborhood. This means that the process similar to the filtering technique (here understood as averaging the input of the neighboring cell) is included in the update rule.

The Cellular Automata algorithm was selected to solve the formulated optimization problem, but it is worth comparing the results with those obtained using other approaches. The validation of the results for example 1 was carried out using a well-recognized Optimality Criteria method (OC) [52]. The topology optimization based on the OC approach was performed simultaneously with the calculations using the CA approach for the test structures shown in Figure 2b and Figure 6a,c in order to compare the computational efficiency. The results are presented in Table 1.

As can be seen, the CA algorithm performs almost as well as the OC method. The CA method has the advantage of producing more accurate results for more complex periodicity schemes, whereas the OC method produces higher final values of compliance. Alternatively, the parameters of the CA method may be adjusted for each individual case to improve the algorithm behavior for the selected, individual example. The objective of this paper was to utilize one set of parameters that achieve satisfactory results for all examples, thereby avoiding the necessity for additional parameter tuning.

## 5. Conclusions

This paper investigates a topology optimization of structures under periodicity constraints with particular emphasis on the structures under self-weight loads. Periodic topology optimization is a cutting-edge approach that allows for the design of lighter and stronger constructions while also considering cost-effective manufacturing. The simple and efficient heuristic Cellular Automata method has been adopted to optimize the process.

In conclusion, this paper presents an original formulation of the problem of periodic topology optimization involving self-weight loads. The solutions obtained demonstrate the algorithm’s capacity to establish connections between subdomains, which is a crucial aspect of designing this type of structure. The algorithm was adapted by determining a single set of parameter values, which enables the solution of the considered tasks. This approach facilitates the acceleration of calculations by reducing the process of parameter adjusting in the method.

The proposed CA approach applied to topology optimization provides fast convergence of the optimization process and complete final results without the so-called grayscale and checkerboard effects. Numerical two- and three-dimensional examples are provided to demonstrate the effectiveness of the proposed method for designing periodic multi-component constructions. The synergy between periodic topology optimization and Cellular Automata opens up new possibilities for designing efficient and innovative structures across different engineering disciplines.

The challenge addressed by this paper concerns numerical examples of periodic topology optimization of massive structures, where the weight of the structure may have a significant impact on the structural property. The considering examples demonstrate the effect of applying mixed loads (i.e., external and self-weight load) on final, optimized topologies. In particular, the rare effect of a negligible impact is also illustrated.

The design of optimal periodic structures under self-weight load is a novel but promising area of research; therefore, further investigation and development of this topic are essential to advance the field. Further development of the topic is required to analyze and validate the results with other optimization methods, particularly for more complex cases such as higher complexity of periodization schemes, periodization for irregular geometries, or optimization with multiple loading schemes.

## Figures and Tables

**Table 1:** Validation of the results. Final values of compliances [Nmm] obtained using the Cellular Automata method and Optimality Criteria method for test structures presented in Figure 2b and Figure 6a,c.

Test Structure	Cellular Automata Method	Optimality Criteria Method
Figure 2b	17,936	17,751
Figure 6a	21,814	21,760
Figure 6c	30,829	32,351

## Data Availability

The data presented in this study are available upon request from the corresponding author.
